# Predictors of the early introduction of solid foods in infants: results of a cohort study

**DOI:** 10.1186/1471-2431-9-60

**Published:** 2009-09-22

**Authors:** Jane A Scott, Colin W Binns, Kathleen I Graham, Wendy H Oddy

**Affiliations:** 1 Nutrition and Dietetics, School of Medicine, Flinders University, Adelaide, Australia; 2 School of Public Health, Curtin University of Technology, Perth, Australia; 3 Telethon Institute of Child Health Research, Perth, Australia

## Abstract

**Background:**

The early introduction of solid foods before 4 months of age has been associated with an increased risk of diarrhoea in infancy and a greater risk of wheeze and increased percentage body fat and weight in childhood. The purpose of this study was to identify the level of compliance with national recommendations related to the timing of the introduction of solid foods and to describe the maternal and infant characteristics associated with the timing of the introduction of solids.

**Methods:**

Subjects were 519 participants in the second longitudinal Perth Infant Feeding Study (PIFS II) recruited from two maternity hospitals in Perth, Western Australia in 2002/3. Data collected prior to, or shortly after discharge from hospital, and at 4, 10, 16, 22, 32, 40 and 52 weeks postpartum included timing of the introduction of solid foods and a variety of maternal and infant characteristics associated with the introduction of solid foods. Multivariate logistic regression was used to identify those factors associated with the risk of introducing solid foods early, which for the purposes of this study was defined as being before 17 weeks.

**Results:**

The median age of introduction of solid foods was 17.6 weeks. In total, 44% of infants had received solids before 17 weeks and 93% of infants had received their first solids before 26 weeks of age. The strongest independent predictors of the early introduction of solids were young maternal age, mother smoking prior to pregnancy and not fully breastfeeding at 4 weeks postpartum. In general, mothers introduced solids earlier than recommended because they perceived their baby to either need them or be ready for them.

**Conclusion:**

This study showed a high level of non-compliance among Australian mothers with the infant feeding recommendation related to the timing of solids that was current at the time. In order to improve compliance health professionals need to be aware of those groups least likely to comply with recommendations and their reasons for non-compliance. Infant feeding recommendations need to be evidence-based, uniformly supported by professionals and widely, clearly and consistently articulated if higher rates of compliance are to be achieved in the future.

## Background

Since 2001 the World Health Organization (WHO) has recommended that infants be exclusively breastfed for 6 months and that complementary foods, including solid foods, be introduced thereafter. The current WHO infant feeding recommendations have the primary aim of reducing morbidity in developing countries [1] and are based on the findings of a systematic review [2], which concluded that there was no significant difference in growth between infants exclusively breastfed for 3-4 months versus 6 months and that later introduction of solids was associated with reduced gastrointestinal infection as reported in a Belarusian study [3].

While the early introduction of solids before 4 months of age has been associated in industrialised populations with an increased risk of diarrhoea in infancy [4] and a greater risk of wheeze and increased percentage body fat and weight in childhood [5], there is considerable debate [6-8] as to whether sufficient evidence exists to support the change from the earlier WHO recommendation [9] that solids be introduced after the 4^th ^month but before the 6^th ^month of life. Prior to 2003 the Australian recommendation with regard to age of introduction of solid foods was the same as the earlier WHO recommendation [10]. Following the release of the current WHO infant feeding recommendations the National Health and Medical Research Council of Australia, in recognition that there is some need to accommodate individual variation, recommended in 2003 that "Exclusive breastfeeding until around six months should be the aim for every infant"[11] More recently in 2008, the European Society for Pediatric Gastroenterology, Hepatology and Nutrition (ESPGHAN) Nutrition Committee recommended that "complementary feeding (i.e. solid foods and liquids other than breast milk or infant formula and follow-on formula) should not be introduced before 17 weeks and not later than 26 weeks"[12]

While much is known about the determinants of breastfeeding initiation and duration, relatively little is known about factors associated with the timing of the introduction of solid foods, particularly with regard to Australian infants. The purpose of this study was to investigate the introduction of solids among a population of Australian infants and to describe the maternal and infant characteristics associated with the timing of the introduction of solids.

## Methods

This present study analyses data from the second Perth Infant Feeding Study (PIFS II) conducted between mid-September 2002 and mid-July 2003, which has been described in detail previously. [13-15] In brief, mothers of healthy infants were recruited from two public hospitals in Perth within the first 3 days postpartum. Mothers were recruited from public hospitals in a deliberate attempt to recruit mothers from socio-economically disadvantaged groups often under-represented in studies of this kind. [16] As a consequence this sample is not representative of women delivering in private hospitals and therefore all births in Perth at the time of this study. Data were obtained from a self-administered baseline questionnaire completed prior to, or shortly after discharge from hospital and from regular telephone follow-up interviews conducted at 4, 10, 16, 22, 32, 40 and 52 weeks postpartum. At each interview information was collected on infant feeding practices including breastfeeding, the use of infant formula and the introduction of complementary foods including other fluids and solid foods.

### Outcome measure - Age of introduction of solids

The age of the infant at the time that the first solid foods were introduced was recorded in weeks. Early introduction of solids was considered to be before 17 weeks of age, which reflected the Australian recommendation that solids be introduced after the 4^th ^month but before the 6^th ^month of life, which was current during the study period [10].

### Exposure measures

A variety of maternal and infant characteristics known or suspected to be associated with the age of introduction of solids were investigated. Infant related variables included gender, birth weight and whether the infant had been admitted to the Special Care Nursery (SCN), infant feeding method at discharge and at 4 weeks of age. Maternal variables included age, level of education, marital status, country of birth, parity, smoking prior to pregnancy, whether she had returned to work by 12 months postpartum or earlier and her infant feeding attitude score, as measured by the Iowa Infant Feeding Attitude Scale (IIFAS). [17]

The IIFAS is a 17 item scale which measures attitudes towards both breast and formula feeding and has been shown previously to be a valid and reliable measure of infant feeding attitudes amongst women in the USA [17] and Scotland [18]. Each item is measured on a 5-point scale and total scores could range from 17 (reflecting positive formula feeding attitudes) to a high of 85 (indicating attitudes that favour breastfeeding). For the purposes of this analysis subjects were categorised according to whether their score was below the sample median score (<65) or equal to or greater than the sample median score (=65).

### Statistical analysis

Data were entered and analysed using the Statistical Package for Social Sciences, Version 17 (SPSS for Windows, SPSS Inc., Chicago, IL, USA). Univariate logistic regression was used to explore the relation between introduction of solids before 17 weeks and each individual explanatory factor. All variables were included in a multivariate logistic regression model to determine which variables were independently predictive of the introduction of solids before 17 weeks of age. Variables found to have a non-significant effect on the model were subsequently removed in a backwards stepwise fashion (p for removal < .05). All variables remaining in the final model were variables for which when excluded the change in deviance compared with the corresponding *X*^2 ^test statistic on the relevant degrees of freedom was significant.

### Ethical considerations

The study was approved by the Human Ethics Committee of the Curtin University of Technology and the Research Ethics Committees of the two participating hospitals. Signed informed consent was obtained from participants. Confidentiality was assured and mothers were advised that their participation was voluntary and that they could withdraw at any time without prejudice.

## Results

In total, 870 of the 1068 women eligible to participate were contacted and 587 completed baseline questionnaires, representing 68% of women contacted. There were no significant differences in the age or level of education of participants compared to women who declined to participate [13]. Data on the age at which infants first received solid foods were available from the follow-up interviews for 519 (88%) of the 587 PIFS II subjects.

### Timing of introduction of solids

**Figure 1:**
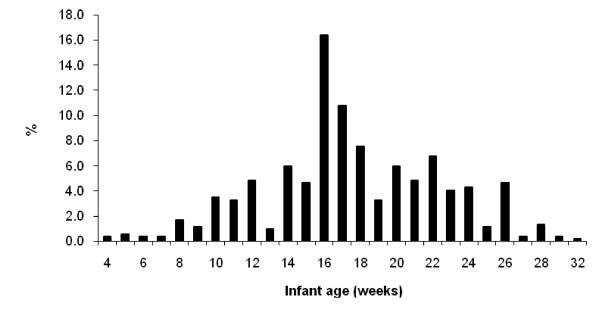
**The distribution of age at which solid foods were first introduced**.

The median age of introduction of solid foods was 17.6 weeks (IQR 15,21) with a frequency peak at 16 weeks (Figure 1). In total, 44% of infants had received solids before 17 weeks (Table 1) and 93% of infants had received their first solids before 26 weeks of age.

**Table 1:** Number (percentage) and univariate odds ratios (95% confidence intervals) for the introduction of solid foods before 17 weeks

**Variable**	**Solids introduced** **<17 weeks**		**Solids introduced** **≥ 17 weeks**		**OR**	**95% CI**
	**N**	**%**	**N**	**%**		
Total (n = 519)	226	43.5	293	56.5		
**Infant factors**						
Sex (n = 519)						
Male	126	44.4	158	55.6	1.08	0.76, 1.52
Female	100	42.6	135	57.4	1.00	
Birth weight (g) (n = 513)						
< 2500	5	41.7	7	58.3	0.70	0.12, 4.23
2500-4499	284	57.7	208	42.3	0.37	0.09, 1.48
≥ 4500	3	33.3	6	66.7	1.00	
Baby admitted to SCN (n = 508)						
Yes	24	45.3	29	54.7	1.06	0.50, 1.89
No	199	43.7	256	55.3	1.00	
**Maternal factors**						
Age (y) (n = 519)						
<20	18	78.3	5	21.7	6.73	2.42, 18.77
20-29	125	49.8	126	50.2	1.78	1.24, 2.56
≥30	86	35.2	158	64.8	1.00	
Years of education (n = 513)						
<12	109	50.9	105	49.1	1.61	1.13, 2.03
≥12	117	39.1	182	60.9	1.00	
Parity (n = 519)						
Multiparous	142	42.6	191	57.4	0.90	0.63, 1.30
Primiparous	85	45.2	102	54.8	1.00	
Marital status (n = 519)						
Married/defacto	205	42.7	27.5	57.3	0.64	0.33, 1.23
Single	21	53.8	18	46.2	1.00	
Mother's country of birth (n = 514)						
Other	14	37.8	23	62.2	0.67	0.34, 1.35
UK/Ireland	17	35.4	31	64.6	0.61	0.32, 1.13
Asia	15	30.0	35	70.0	0.47	0.25, 0.90
Australia/New Zealand	180	47.5	199	52.5	1.00	
Mother's work status (n = 491)						
Returned to work < 6 mo	54	41.2	77	58.8	1.04	0.68, 1.58
Returned to work 6-12 mo	40	51.9	37	48.1	1.60	0.97, 2.66
Not working at 12 mo	114	40.3	169	59.7	1.00	
Mother's infant feeding attitude (n = 519)						
High IIFAS^a ^score	95	34.9	177	65.1	0.48	0.33, 0.68
Low IIFAS score	131	53.0	116	47.0	1.00	
Mother smoked prior to pregnancy (n = 517)						
Yes	112	57.1	84	42.9	2.45	1.70, 3.53
No	113	35.2	208	64.8	1.00	
Feeding method at discharge (n = 519)						
Formula	23	76.7	7	23.3	4.53	1.90, 10.80
Combination	34	39.1	53	60.9	0.88	0.55, 1.42
Fully breastfed	169	42.0	233	58.0	1.00	
Feeding method at 4 weeks (n = 491)						
Formula	74	62.7	44	37.3	3.47	2.22, 5.44
Combination	43	47.3	48	52.7	1.85	1.14, 2.99
Fully breastfed	92	32.6	190	67.4	1.00	

SCN Special Care Nursery

OR = Odds Ratio

95%CI = 95% confidence interval

IIFAS = Iowa Infant Feeding Attitude Score Range from 17 -- 85 with a high score indicative of positive breastfeeding attitudes. Scores dichotomised to low score below median score for study sample (<65), high score median or above (≥ 65).

**Figure 2:**
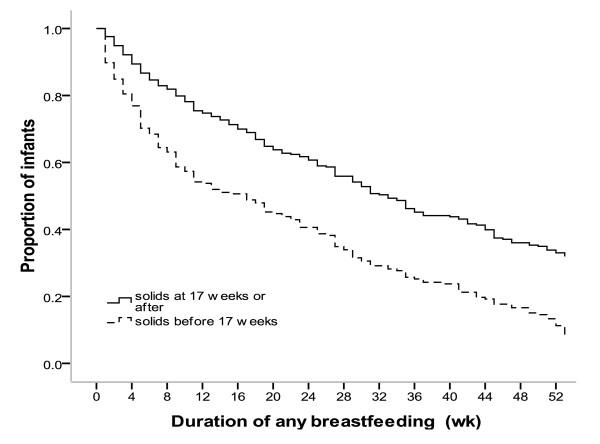
**The association of breastfeeding duration and age of introduction of solid foods**.

There was a significant association between the timing of the introduction of solids and the duration of breastfeeding (log rank test p < 0.001) (Figure 2). Those mothers who introduced solids at or after 17 weeks breastfed an average of 11 weeks longer than mothers who introduced solids before 17 weeks (mean breastfeeding duration 30.7 wk vs 19.7 wk, p < 0.001).

### Factors associated with the timing of solid foods

Univariate analysis revealed that younger, less well educated (<12 years of schooling) women or those who smoked prior to pregnancy were more likely to introduce solids before 17 weeks, as were those women who were formula feeding at discharge from hospital or fully or partially formula feeding at 4 weeks postpartum. On the other hand, Asian mothers and those with a high IIFAS, indicative of positive breastfeeding attitudes, were less likely to introduce solids before 17 weeks (Table 1). Following adjustment for potential confounders, only young maternal age, smoking prior to pregnancy and not fully breastfeeding at 4 weeks postpartum were found to be independently associated with the introduction of solids before 17 weeks (Table 2).

**Table 2:** Factors independently^a ^associated with the introduction of solid foods before 17 weeks (n = 440)

	**Mean age of introduction of solids (weeks)**	**AdjOR**	**95% CI**
Maternal age (yrs)			
<20	14.2	4.25	1.21, 14.93
20-29	17.0	1.40	0.02, 2.12
>30	18.7	1.00	
Mother smoked prior to pregnancy			
Yes	16.0	1.91	1.26, 2.90
No	18.7	1.00	
Feeding method at 4 weeks			
Fully formula fed	15.5	2.61	1.48, 4.61
Partially breastfed	17.0	1.80	1.02, 3.13
Fully breastfed	18.9	1.00	

a Non-significant factors included infant sex, birth weight and whether the infant had been admitted to the Special Care Nursery (SCN), infant feeding method at discharge, mother's level of education, marital status, country of birth, parity, whether she had returned to work by 12 months postpartum and her infant feeding attitude score

b All variables in the final model were variables for which when excluded the change in deviance compared with the corresponding *X*^2 ^test statistics on the relevant degrees of freedom was significant.

^C^Adjusted odds ratios were derived from the logistic regression coefficients.

### Reasons for introducing solid foods before 17 weeks

The main reasons that women gave for introducing solids to their infants before 17 weeks were that their baby was hungry or big for their age (55%), that their baby was old enough to start solids (17%), that they used solids to settle the baby or help them sleep through the night (15%) and/or that the baby was showing interest in solids or were in some other way indicating they were ready for solids (12%), for example by putting their hands or other objects in their mouth and chewing on them (Table 3). Younger or primiparous mothers were no more likely to perceive their infant to be hungry than older or multiparous women (χ^2 ^p = 0.396 and 0.433, respectively).

**Table 3:** Main reasons for introducing solids before 17 weeks of age

**Reason for introducing solids**	**Solids before 17 weeks** **(n = 226)**	
	
	**N**	**%**
Baby hungry/large baby	125	55
Baby old enough/to wean baby	39	17
To settle baby/help sleep through the night	34	15
Baby interested^a^/ready	26	12

^a ^Interest indicated by baby putting hands and other objects into mouth and/or chewing hands, and other objects

## Discussion

Although the current Australian infant feeding recommendation is that infants should be exclusively breastfed for around 6 months [11], during 2002-3 when the present study was conducted, Australian recommendations stated that solids foods be introduced during the first 4 to 6 months [10]. Nevertheless, compliance by mothers in this study with the recommendations current at the time was poor, with close to half of all infants (43.5%) receiving their first solid foods before 17 weeks of age. This finding is reasonably consistent with the results of the 2001 Australian National Health Survey [19] which indicated that 55% of infants over 18 weeks were receiving solids.

Non-adherence to feeding recommendations is not limited to Australian mothers. Even higher rates of non-compliance with the earlier WHO recommendation were reported in a number of studies of industrialised populations conducted in the late 1990s and early 2000s, with 45% of New Zealand [20], 63% of Finnish [21] and 70% of Canadian [22] infants receiving solid foods before 4 months of age. Further, a Scottish study conducted in 1999 [23] showed that 40% of infants had received solid foods by 12 weeks of age. There is however, encouraging evidence that infant feeding practices are amenable to change with recent Swiss [24] and US studies [25] reporting that only 5% and 21% of infants, respectively, had been fed solids before 4 months.

We found that mothers who introduced solids at or after 17 weeks breastfed for an average of 11 weeks (2.6 months) longer than mothers who introduced solids before 17 weeks, which is consistent with recent studies in Lithuania [26], Switzerland [24] and the USA [25], all of which reported a 2 month difference in duration of overall breastfeeding.

After controlling for different potential confounders we found significant differences in the timing of the introduction of solids according to different socio-demographic and lifestyle factors. Consistent with earlier studies we found that younger mothers [21,23,24,27,28] and mothers who smoked [24,28,29] were most likely to introduce solids early. Similar to previous studies which have reported a protective association with ever having been breastfed [27,29,30], we found a protective univariate association with breastfeeding at discharge, although this factor did not remain significant in the multivariate analysis. We did find however, that infant feeding method at 4 weeks postpartum was a strong independent predictor of age of introduction of solids, with mothers who were fully breastfeeding at 4 weeks being less likely to introduce solids before 17 weeks than mothers who were either fully or partially formula feeding their infant at this age. A similar association has previously been reported in a study of Italian infants [29] which reported that mothers who introduced formula within 1 month were almost 3 times as likely to have introduced solid foods early compared with mothers who never supplied their infant with formula. Unlike earlier studies we found no independent association between the timing of solids and parity [30,31], maternal level of education [27,28,30] and mothers in this study were no more likely to introduce solids earlier to boys than girls [21,23,28]. However, we did find in the univariate analysis that women with less than 12 years of education were significantly more likely to introduce solids before 17 weeks than those with more than 12 years of education. As such our results identify less educated women as a potential target group for future education and support programs.

The main reason in this study that mothers introduced solids to their infants before 17 weeks was that they perceived that their infant either needed solids or that they were ready for them. In both instances need and readiness for solids was determined by relatively subjective indicators such as "hunger" or the putting of hands and other objects into the infant's mouth. Wright et al [4] in a study of English women also reported that the strongest perceived influence on the early introduction of solids was the belief the baby was hungry. Furthermore women were acting on their own initiative and intuition as few cited written or professional influences. This was the case also in this study with a number of women stating that their baby was '*old enough*' or was '*ready for solids*', with very few (<5%) indicating that they were advised by a health professional. Future interventions that aim to reduce the early introduction of solid foods need to educate mothers how to interpret infant behaviours and what to do if an infant seems hungry or unsettled. As neither maternal age nor parity was associated with perception of infant hunger such programs should be targeted at all women and not just young or first time mothers.

While this study did not investigate how a mother determined that her infant was hungry, an earlier qualitative study of Scottish women identified a number of subjective characteristics perceived by mothers to indicate that a child was hungry, for example '*baby having a hungry cry*', '*looking for milk*', '*chewing hands*' and the baby looking at the food others are eating [32]. As in this current study, a main aim of introducing solids identified by the Scottish mothers was '*to settle the baby so that the baby was more contented and happier*'.

The eating of solids is seen by many mothers as a developmental milestone and a number of women in the earlier Scottish study indicated they experienced a sense of pride when their baby first had solid food [32]. As for all developmental milestones, mothers are often keen for their child to achieve this milestone, and a number of women in this current study (~5%) indicated that they first gave their baby solids to see if they were interested or to get them used to different tastes and textures, suggesting in these cases that the introduction of solids was mother-led and not infant-led.

Compliance with feeding recommendations assumes that women are both aware of and in agreement with the recommendations of the time. Alder et al. [23] reported that Scottish women were significantly more likely to have introduced solids by 12 weeks if they disagreed with the then current recommendation to delay giving solids until 4 months. Both a lack of awareness and agreement is likely to occur if the reasons for feeding recommendations are not clearly articulated or uniformly supported. The ongoing debate over the appropriateness of the current WHO infant feeding recommendations with regards to the timing of the introduction of complementary feeding [6-8] can only add to any existing confusion on the part of mothers.

A limitation of this study was that less than 60 percent of eligible women participated. Nevertheless, the sample size of the study was relatively large and there was no significant difference in maternal age and level of education between participants and those declining to participate, suggesting that the sample was representative of the population from which it was drawn. However, as the sample was drawn from two public hospitals the results do not necessarily reflect the practices of Perth women from higher socio-economic groups who deliver in private hospitals. The study had a number of strengths; firstly data were collected prospectively at birth and seven additional time points over a 12 month period thereby reducing the likelihood of maternal recall bias and the potential for "heaping of data" [33] in relation to events of interest. Secondly, the timing of the introduction of solids was measured in weeks, allowing the time to event to be precisely measured and thirdly, we clearly defined early introduction of solids as being before 17 weeks. In many studies infant age is poorly defined, and while early introduction of solids is defined commonly as being before 4 months it is not clear if this refers to completed months of age and if so how weeks of age are converted to months of age. For instance, Webb et al [34] defined less than 4 months of age as being 0-17 weeks, whereas Grummer-Strawn et al [27] identified infant feeding patterns at 4 months as events occurring in weeks 15 to 18. Furthermore, some studies have used the terms "before 4 months" and "at 4 months" interchangeably [29], creating additional confusion. Lack of a precise and consistent definition of early introduction of solids makes it difficult to compare results across studies. Our results demonstrated a clear peak at 16 weeks of introducing solids which suggests that mothers might calculate months in terms of a four week month with 4 months being interpreted as 16 weeks. The recent recommendation of the ESPGHAN Nutrition Committee [12] that complementary feeding should not be introduced before 17 weeks and not later than 26 weeks is less open to misinterpretation and we recommend that future studies adopt "17 weeks of age" and "26 weeks" as the definition of 4 and 6 months, respectively.

## Conclusion

This study showed a high level of non-compliance among Australian mothers with the infant feeding recommendation that solid foods be introduced after 17 weeks, which was the National infant feeding guideline current at the time of this study. If a high level of compliance is to be achieved then health professionals need to be aware of those groups least likely to comply with recommendations and their reasons for non-compliance. Future health promotion programs need to educate mothers how to interpret infant behaviour and what to do if their infant seems hungry and infant feeding recommendations need to be evidence-based, uniformly supported by professionals and widely, clearly and consistently articulated.

## Abbreviations

Adj OR: Adjusted Odds Ratio; IIFAS: Iowa Infant Feeding Attitude Scale; IQR: Inter Quartile Range; OR: Odds Ratio; SCN: Special Care Nursery; 95% CI:95% Confidence Interval.

## Competing interests

The authors declare that they have no competing interests.

## Authors' contributions

JAS contributed to the design of the study, analyzed the data for this manuscript and wrote the first draft of the manuscript, CWB conceived the idea for the study, contributed to the design of the study and revised drafts of the paper, KIG coordinated the project and revised drafts of the paper, WHO contributed to the design of the study and revised drafts of the paper

## Pre-publication history

The pre-publication history for this paper can be accessed here:


